# Insights of organic fertilizer micro flora of bovine manure and their useful potentials in sustainable agriculture

**DOI:** 10.1371/journal.pone.0226155

**Published:** 2019-12-20

**Authors:** Dalaq Aiysha, Zakia Latif

**Affiliations:** Department of Microbiology and Molecular Genetics, University of the Punjab, Lahore, Pakistan; Gifu University, JAPAN

## Abstract

Exploration of diverse environmental samples for plant growth-promoting microbes to fulfill the increasing demand for sustainable agriculture resulted in increased use of bacterial biofertilizer. We aimed for the isolation of plant growth-promoting as well as antibiotic sensitive bacteria from bovine manure samples. The basic theme of our study is to highlight potentials of bacteria in manure and the unchecked risk associated with the application of manure i.e. introducing antibiotic-resistant microbial flora, as fertilizer. Fifty-two, morphologically distinct isolates; from eight different manure samples, were subjected to plant growth-promoting parametric tests along with antibiotic resistance. Thirteen antibiotic sensitive bacterial strains with potentials of plant growth promotion further characterized by 16S rRNA ribotyping and the identified genera were *Stenotrophomonas*, *Achromobacter*, *Pseudomonas*, and *Brevibacillus*. Successful radish seeds germination under sterile in-vitro conditions showed the potential of selected bacterial isolates as plant growth-promoting bacteria. The results of this study confirmed plant growth-promoting characteristics of bovine manures’ bacterial strains along with an alarming antibiotic resistance load which comprises 75% of bacterial isolated population. Our study showed distinct results of un-explored manure bacterial isolates for plant growth promotion and flagged ways associated with unchecked manure application in agriculture soil through high load of antibiotic resistant bacteria.

## Introduction

Animal manure is undigested components of animal feed, enriched with 55 to 95% of nitrogen and 70% of phosphorus ingested, excreted in manure [1]. The livestock products are macro- and micronutrient enriched sources and supplies 18% food energy [2,3]. The application of manure in agricultural land provides several benefits like nutrient recycling, better crop productivity, and improved organic matter [4]. Indirectly reduced soil erosion and increased water holding capacity improves soil water availability for cultivation [5]. In an eight-year field study [6] of applying chemical fertilizers (NPK) and manure mixed chemical fertilizers explores agricultural benefits of comparative fertilization. Application of NPK-manure mixes increased soil fertility, crop yield over non-manure NPK mix fertilization. Applying inorganic fertilizers in agricultural lands impose direct negative effects due to the underlying mechanism of osmotic effect induced by fertilizer itself. This direct negative effect alters soil pH [7]. Organic manure fertilization has a positive effect on soil microbial communities [8–10]. Therefore, applying dairy manure in cultivation land is an established practice for nutrient recycling and improving organic matter. This indirectly improves soil quality and field performance [11].

Livestock production is the most valuable part in the lives of millions across the globe [12]. This practice is not beneficial in all aspects of the association. The major environmental challenges, eutrophication, biodiversity loss, acidification, and global warming are at an exponential rate due to increased livestock production. Apart from physical environmental effects, public health is also at risk [13]. As per WHO, antibiotic resistance is a major public health issue worldwide. As proposed by McKenna (2014), deaths due to antibiotic resistance will be ten million per year globally [14]. Such an alarming threat to public health linked with the increased use of antibiotics in animal and human feed. The ecological spread of antibiotic resistance is interrelated with animal wastes alone. This spread is due to the environmental wastes of dairy farms carrying pathogenic and non-pathogenic antibiotic resistant microbial populations [15]. Such an unchecked release of animal wastes imposes a direct potential threat to human health [16]. Organic wastes, including animal manure and sewage sludge, are commonly used as fertilizer in agricultural lands. Such organic fertilizer contains a high amount of antibiotic resistant bacteria. The application of manure alone in agriculture land exemplifies a direct addition of antibiotic resistance microflora in our food chain [17–19].

Microbes in the manure are impassively introduced into the soil by water fluxes [20] and by motility of microbial communities but to shorter distances. Soil contamination with antibiotic resistance primarily depends upon the type and survival rate of manure microbial flora [21]. It is reported in a study that manure application as fertilization of agricultural soil showed a more diversified microbial population as compared to non-manure fertilized soil. This thirteen-year manure fertilized soil microbial analysis does not provide the positive or negative microbial population of microbes introduced by manure itself [22]. Studies reported the positive effect of nutrient distribution associated with manure application in cropland and related increased harvest [23] even in acidic soil conditions [6]. Therefore, eliminating the application of manure for its negative effect i.e. introduction of the antibiotic resistant microbial population does not justify its positive aspects in agricultural practice. To our knowledge, isolation of plant growth-promoting bacteria mostly isolated from soil [24,25]. The fundamental aspect of our study is to isolate culturable bacterial strains from different manure samples of local bovine family breeds and in-vitro characterization for plant growth-promoting traits and antibiotic susceptibility profile to check their effect on radish germination.

## Results

A total of fifty-two bacterial colonies were selected by serial dilution technique from eight different animal dung samples (Table 1). Samples of Nili Ravi (Water buffalo) dung from the village farm resulted in the maximum number of bacterial isolates while the sample of cow dung from town farm resulted in the least number of strains. Colony-forming units (CFU) were also analyzed representing bacterial population size/gram of fresh sample.

**Table 1:** Bacterial population size of different bovine animals.

Serial No.	Bovine animal	No. of selected isolates	CFU/mg of sample
1	L. Cow	1	2.0 × 10^6^
2	L. Ox	5	3.9 × 10^7^
3	L. Water buffalo	7	4.0 × 10^6^
4	L. Calf	3	4.4 × 10^6^
5	K. Cow	12	6.0 × 10^6^
6	K. Ox	6	5.3 × 10^7^
7	K. Water buffalo	14	7.3 × 10^9^
8	K. Calf	4	3.0 × 10^6^

Sample area: L, Lahore; K, Khanqah Dogran

### 1. Metabolic profile

**Fig 1:**
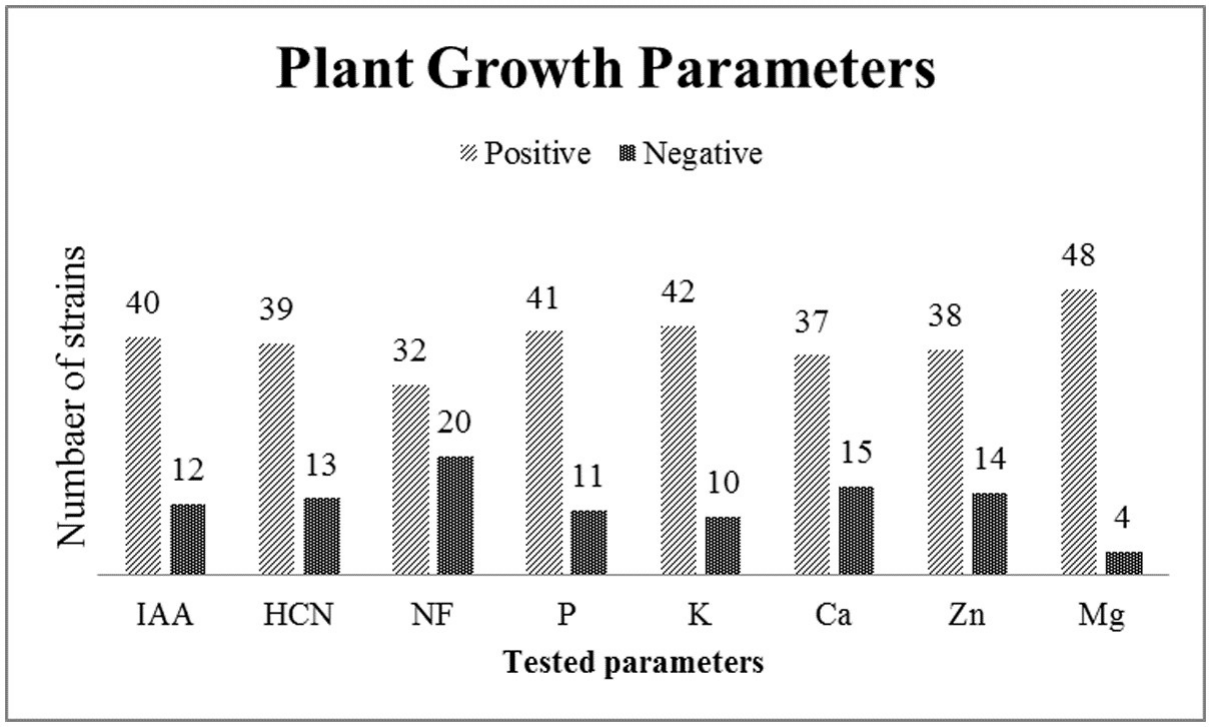
Major plant growth promoting parameters of isolated bacterial strains (n = 52). NF = nitrogen fixation, IAA = indole acetic acid production, HCN = hydrogen cyanide production, P = phosphorus solubilization, K = potassium solubilization, Ca = calcium.

Analysis of metabolic profile includes plant growth-promoting factors (IAA and NF), antifungal test (HCN) and solubilization of insoluble minerals essential for plant growth was performed. A variable number of bacterial strains exhibited (Fig 1), among total numbers (n = 52), different responses for each test essential for plant growth.

#### a. Nitrogen fixation

The ability of the isolates to fix nitrogen was analyzed using a nitrogen-free medium. The growth of thirty-two (61%) bacterial isolates on this medium indicated to exhibit the potential to fix nitrogen, whereas twenty (39%) isolates lack this ability (Fig 1).

#### b. Auxin production

**Fig 2:**
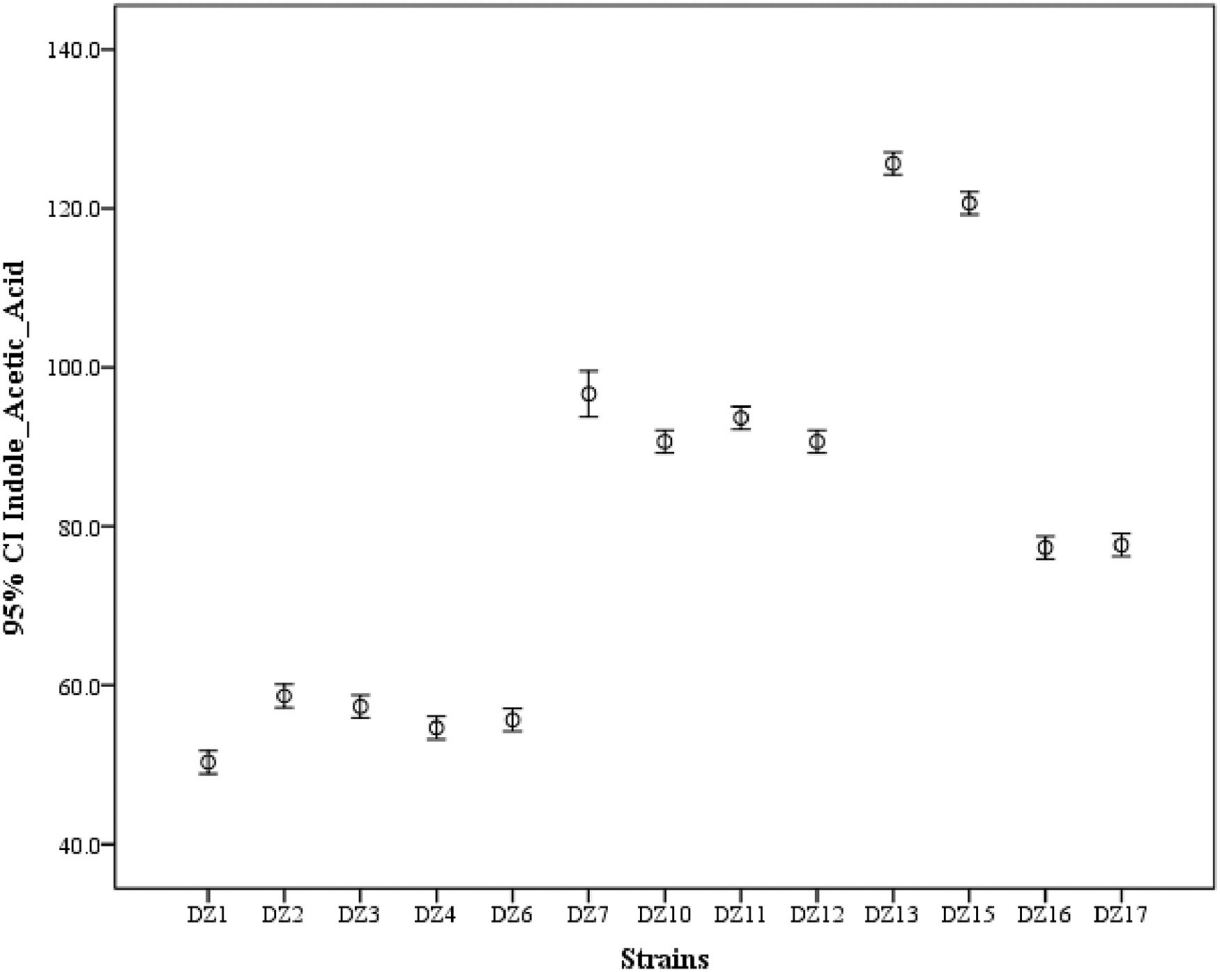
Indole acetic acid (IAA) production ability of selected bacterial strains. Data represented as means and standard error.

Isolates’ ability for Indole acetic acid (IAA) production was examined spectrophotometrically at 535nm and compared according to the IAA standard curve. As shown (Fig 1), forty (77%) out of fifty-two isolates were positive for IAA production and only twelve (23%) of the isolates negative. Results in the graph (Fig 2) showed that the highest (126 μg/ml) IAA production was observed by DZ.13 while the lowest (50 μg/ml) was observed by DZ.1.

#### c. HCN production

Hydrogen cyanide (HCN) production by bacterial isolates was examined and recorded results accordingly. It was found (Fig 1) that thirteen (25%) were negative for HCN production and thirty-nine (75%) isolates could produce HCN as filter paper color was changed to orange-brown.

#### d. Phosphorus and potassium solubilization

**Fig 3:**
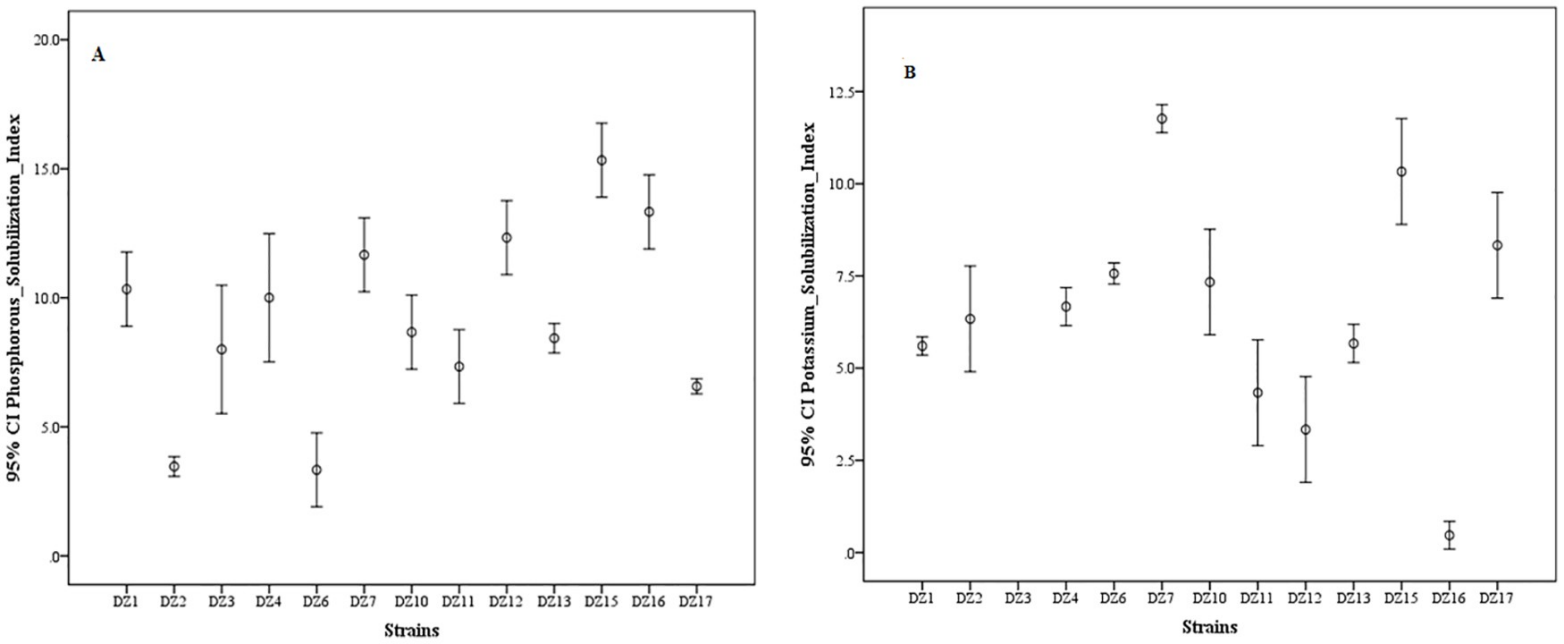
Phosphate (A) and potassium (B) solubilization index of sorted bacterial strains (n = 13). Data represented as means and standard error.

Phosphorus solubility was analyzed using the NBRIP medium and the formation of halo zones around inoculated strains indicated their respective ability. A total of forty-one (79%) bacterial isolates showed phosphorus solubilizing ability while eleven (21%) isolates lack this ability (Fig 1). Among the selected thirteen strains highest phosphate solubilization index (15) was observed by DZ.15 and lowest (3) by DZ.2 (Fig 3a). Potassium solubilization was determined by measuring a clear zone around growth which indicated their ability to solubilize potassium. Forty-two (81%) of the isolates were positive for potassium solubilization and ten (19%) were negative for potassium solubilization (Fig 1). Among the selected thirteen strains highest potassium solubilization index (13) was observed by DZ.3 and lowest (0.5) by DZ.16 (Fig 3b).

#### e. Calcium, magnesium and zinc solubilization

**Fig 4:**
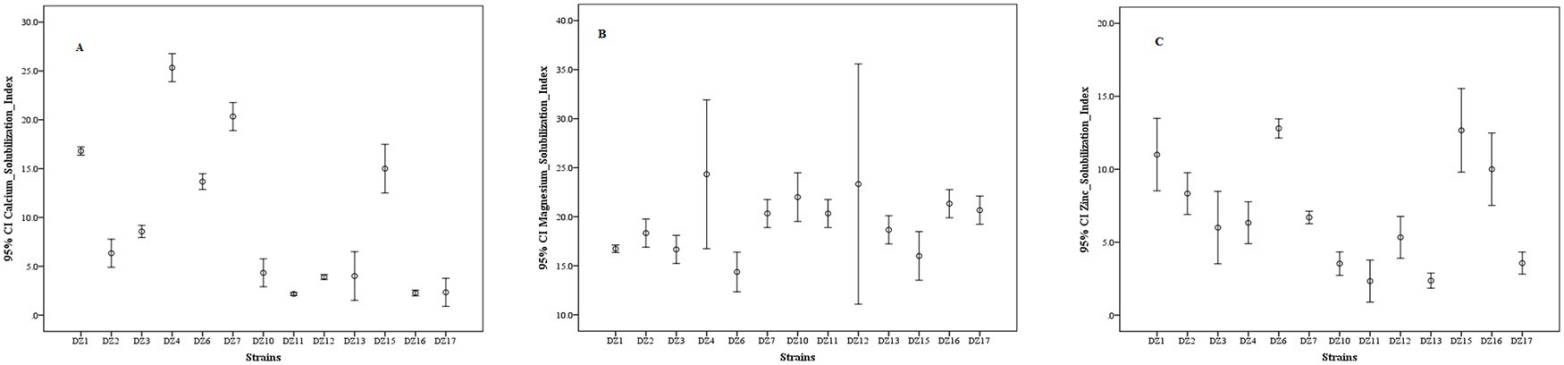
Calcium (A), magnesium (B) and zinc (C) solubilization index of sorted bacterial strains (n = 13). All experiments were performed in triplicates and data represented as means and standard deviation.

Calcium solubilization by isolates was measured by halo zone formation and thirty-seven (71%) showed positive results while fifteen (29%) were negative (Fig 1). Among the selected thirteen strains highest calcium solubilization index (25) was observed by DZ.4 and lowest (2) by DZ 11 (Fig 4a). Magnesium solubilization by all isolated strains revealed that 48 (92%) out of 52 were positive and only 4 (8%) were negative (Fig 1). Among the selected thirteen strains highest magnesium solubilization index (24) was observed by DZ.4 and lowest (14) by DZ.6 (Fig 4b). Zinc solubilization of the isolates was tested and thirty-eight (73%) showed positive results while fourteen (27%) were negative in agar plate assay (Fig 1). Among the selected thirteen strains highest zinc solubilization index (12.8) was observed by DZ.6 and lowest (2.3) by DZ.11 (Fig 4c). All nutrient uptake abilities indicated the potential benefits of using bacterial isolates as biofertilizer.

### 2. Antibiogram assay

**Fig 5:**
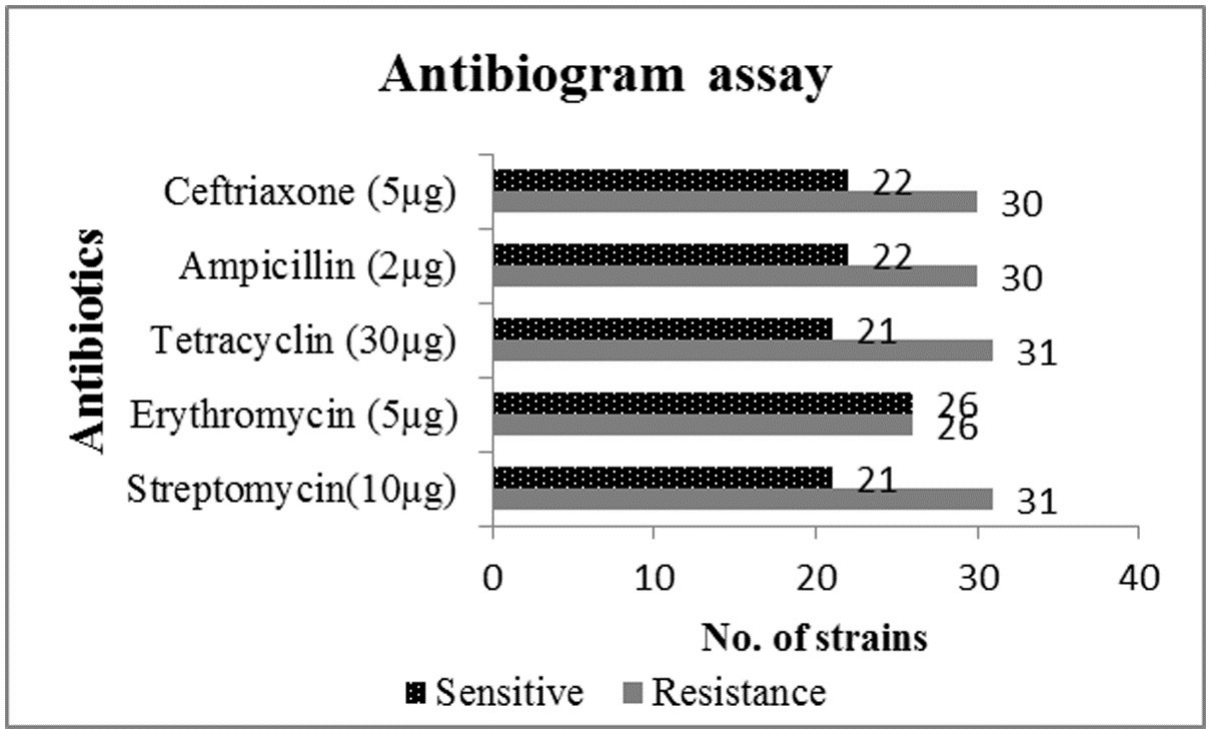
Antibiotic resistance and sensitivity of bacterial isolates against selected antibiotics.

Isolated bacterial strains showed variable resistance against selected antibiotics by the disc diffusion method. Out of a total of 52 isolates 42%, 42%, 41%, 50%, and 41% were resistant to ceftriaxone, ampicillin, tetracycline, erythromycin, and streptomycin, respectively (Fig 5). Among the bacterial isolates, only 13 showed sensitivity to tested antibiotics.

### 3. Molecular characterization of selected bacterial strains

**Fig 6:**
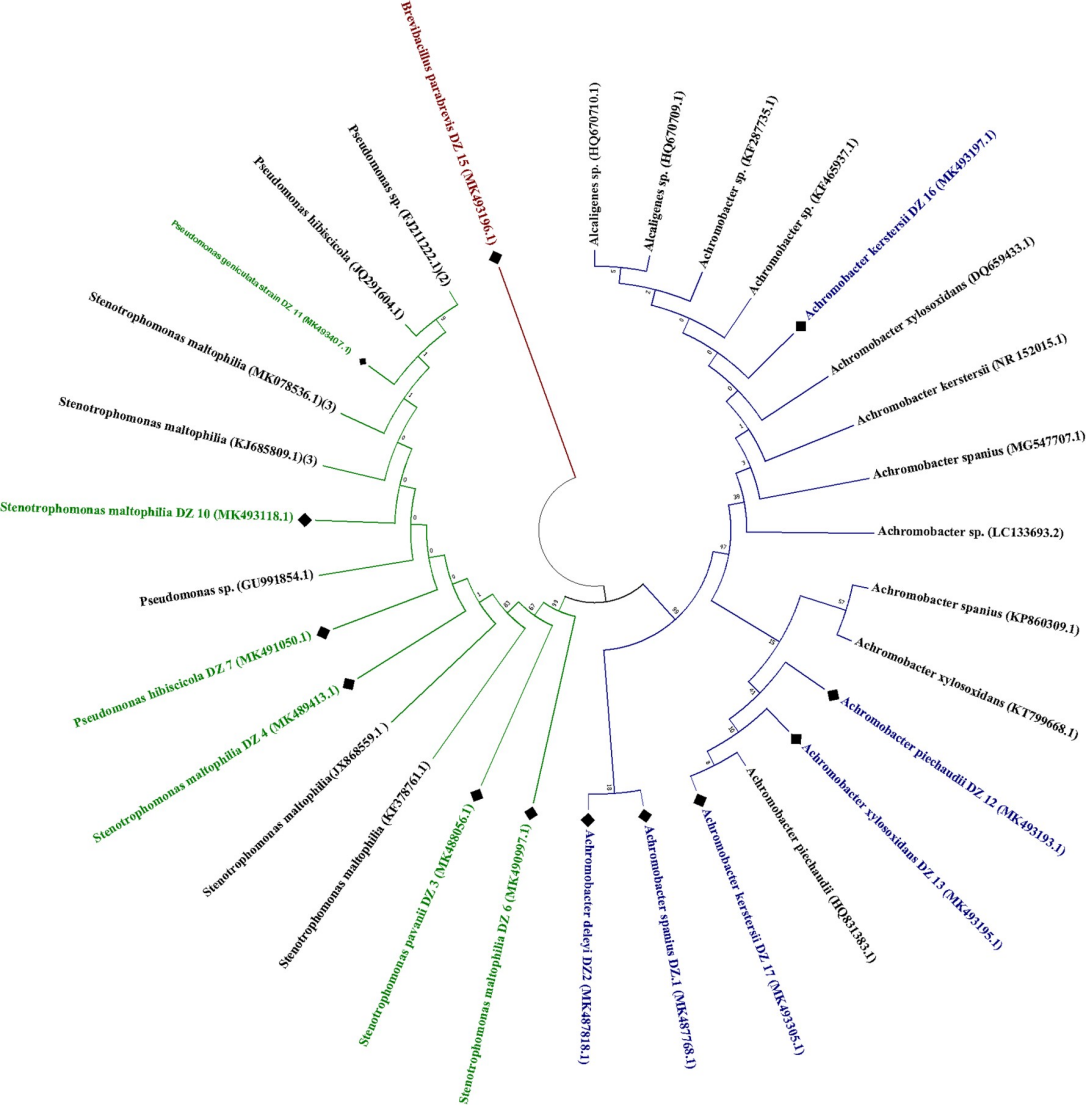
Phylogenetic tree constructed using 16S rRNA gene sequence analysis. Maximum likelihood tree showing the evolutionary relationship between sorted potential plant growth promoting bacterial strains and previously reported strains having highest similarity index from NCBI GenBank.

In the comparison of the metabolic profile of all bacterial strains, thirteen strains exhibiting significant characteristic features were selected for molecular identification using 16S rRNA ribotyping. The sequences were submitted to GenBank for accession numbers (Table 2). Constructed dendrogram distinguished selected strains in four genera due to its high similarity and proximity, i.e., (i) *Strenthomonas* sp.; (ii) *Pseudomonas* sp.; (iii) *Achromobacter* sp. and (iv) *Brevibacillus* sp. Statistical analysis of constructed tree (Fig 6), generated by the maximum likelihood method, by bootstrapping (100) revealed a similarity index of all isolated strains. These strains were broadly categorized into three different classes. Strain DZ.15 *Brevibacillus parabrevis* (accession number MK493196) showed no similarity to all other isolated strains. The second clad included six isolates; DZ.1, DZ.2, DZ.12, DZ.13, DZ.16, DZ.17, belonging to *Achromobacter* genera i.e., *A*. *spanius*, *A*. *deleyi*, *A*. *piechaudii*, *A*. *xylosoxidans* and *A*. *kerstersii* for both last isolates i.e. DZ.16 and DZ.17 respectively (Table 2). In this clade DZ.1, DZ.2 has 99% similarity with each other, whereas these have 97% similarity with DZ.12 and all these have 90% similarity with rest three isolates; DZ.13, DZ.16, DZ.17. The third clade in the tree includes five isolates, where DZ.11 has 90% similarity with the rest of the clad. While, DZ.10 showed 92% similarity and all rest i.e. DZ.10, DZ.7, DZ.6, DZ.4, and DZ.3 have 99% similarity with each other in the clad.

**Table 2:** Molecular identification by 16S rRNA ribotyping and accession numbers of the selected bacterial strains.

Sr.No	Lab ID	16S rRNA gene sequences	Accession No.
1	DZ.1	*Achromobacter spanius*	MK487768
2	DZ.2	*Achromobacter deleyi*	MK487818
3	DZ.3	*Stenotrophomonas pavanii*	MK488056
4	DZ.4	*Stenotrophomonas maltophilia*	MK489413
5	DZ.6	*Stenotrophomonas maltophilia*	MK490997
6	DZ.7	*Pseudomonas hibiscicola*	MK491050
7	DZ.10	*Stenotrophomonas maltophilia*	MK493118
8	DZ.11	*Pseudomonas geniculata*	MK493407
9	DZ.12	*Achromobacter piechaudii*	MK493193
10	DZ.13	*Achromobacter xylosoxidans*	MK493195
11	DZ.15	*Brevibacillus parabrevis*	MK493196
12	DZ.16	*Achromobacter kerstersii*	MK493197
13	DZ.17	*Achromobacter kerstersii*	MK493305

### 4. Radish seed germination in vitro conditions

**Fig 7:**
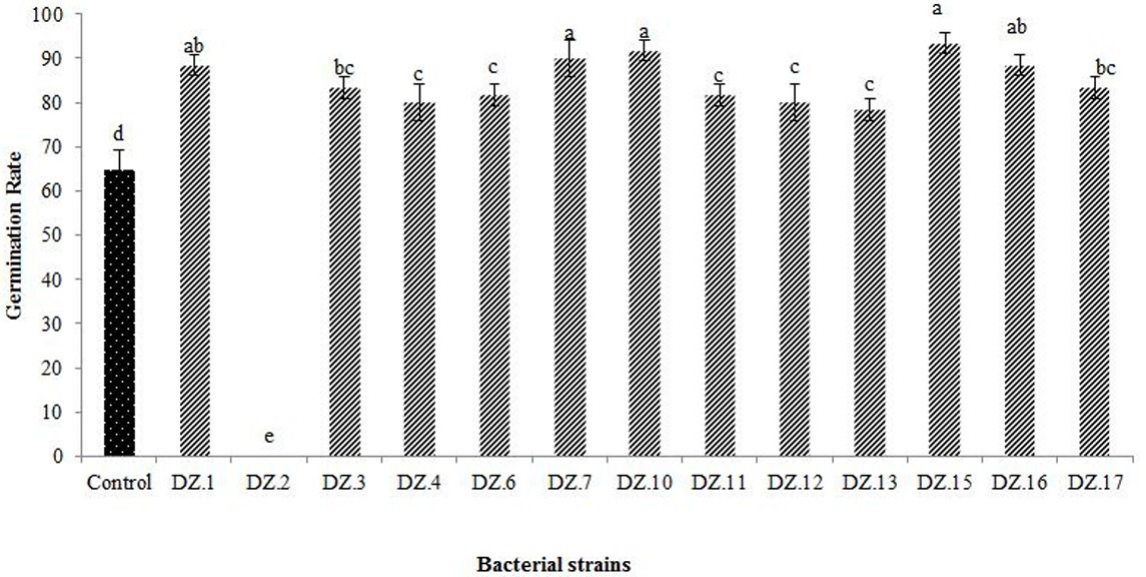
Effect of bacterial isolates on radish seed germination. Error bars are standard deviations from triplicates that received the same treatment. Different letters represent a statistically significant difference between treatments (Duncan’s test, *P* < 0.05).

Radish seed germination experiments showed a significant effect on seed germination rate as compared to the control (Fig 7). One strain (DZ.2) had a deleterious effect of a 100% decrease for the radish germination rate. While rest strains (n = 12) showed a 23% to 46% increase in seed germination rate. The maximum increase in the germination rate was observed with the DZ.15 strain. These results showed that bacterial strains except DZ.2 have potential positive results to enhance plant growth. Statistical analysis of seed germination using ANOVA and DMRT showed that statistical means of bacterial inoculation are variably distinct. There were significant effects of bacterial isolates of bovine manure on seed germination at p<0.05 level for the inoculation [F (13, 28) = 131.462. p = 0.001] except DZ.2.

## Discussion

Backed by centuries’ old practices, we hypothesized that microbial flora of raw, wet bovine manure exhibits maximum capabilities of plant growth promotion. We commonly use cow dung in the sub-continent in the agricultural system as, fertilizer for a crop, a source of energy and pest repellent [26]. Two studies have been reported on cow dung microbial diversity including predominant genera; *Enterobacter*, *Escherichia*, *Klebsiella*, *Kluyvera*, *Morgarella*, *Pasteurella*, *Providencia*, *Pseudomonas*, *Citrobacter*, *Lactobacillus*, and *Corynebacterium* [27, 28].. To our knowledge, none of the reported studies focused on microbial isolation and characterization of the dung bacterial flora of buffalo (Neli Ravi), ox, calf, or cow. Our study is unique as the maximum animals in the bovine family targeted for dung sampling and the isolation of dung microflora. The results obtained from in-vitro experiments i.e. IAA production, putative nitrogen fixation, phosphorus, potassium, zinc, calcium, magnesium solubilization along with HCN production, supports our hypothesis.

**Fig 8:**
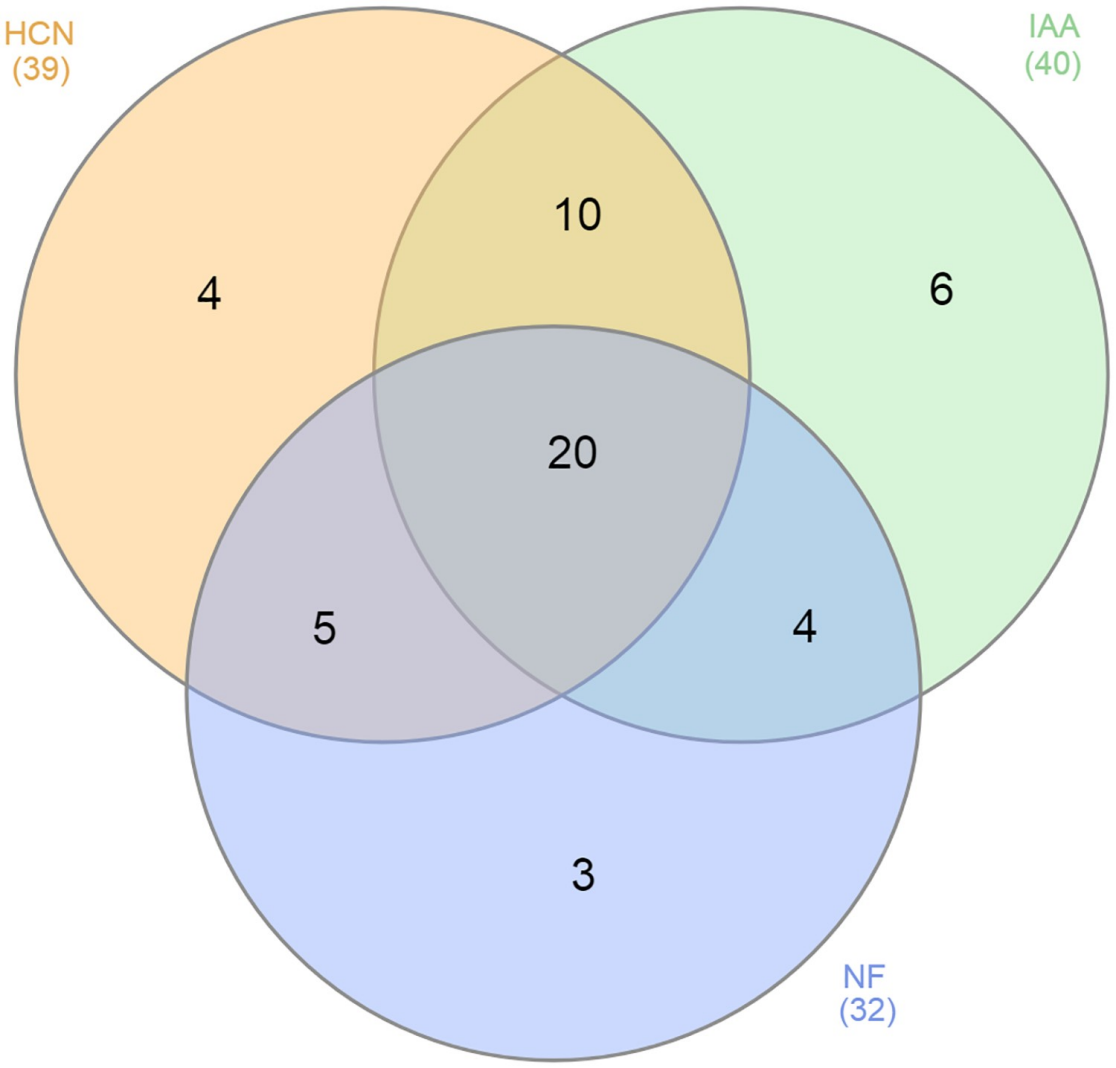
Venn diagram of presumptive NF, IAA and HCN production. Among identified bacterial strains (n = 52); 32 NF positive (61%), 40 IAA positive (77%) and 39 HCN positive (75%) were sorted using Venn diagram.

Venn diagram analysis [29] of putative nitrogen fixation, HCN and IAA production has shown a diversified relation among all positive strains regarding these three tests (Fig 8). It showed that 20 strains were positive for all these PGP traits, 10 positives for HCN and IAA, 5 positives for HCN and nitrogen fixation, 4 positive for nitrogen fixation and IAA production. Well-studied nitrogen-fixing bacteria are *Azotobacter*, *Alcaligenes*, *Azospirillium*, *Acinetobacter*, *Arthrobacter*, *Erwinia*, *Enterobacter*, *Bacillus*, *Rhizobium*, *Flavobacterium*, *Serratia* and *Mycobacterium* [30]. Plant growth-promoting bacteria reported, to our best knowledge, for IAA production are *Rhizobium*, *Pseudomonas*, *Azospirillium*, *Bacillus*, *Azotobacter*, *Alcaligenes*, *Enterobacter*, *Bradyrhizobium* and *Burkholderia* [31], for HCN production are *Pseudomonas*, *Rhizobium*, *Enterobacter*, *Bacillus*, *Aeromonas*, and *Alcaligenes* [32]; our isolated genera had not been reported previously in this regard.

Phosphorous is a second major element after nitrogen for normal plant growth and development [33]. The extraction of phosphorus from commonly available rock phosphorous is cumbersome, energy, and time-consuming process [34–36]. In our study, among isolated bacterial strains seventy-nine percent (n = 41/52) were positive for phosphorous solubilization as showed by halo zone formation around bacterial growth. In our agricultural practices, farmers commonly go for regular use of nitrogen and phosphorus chemical fertilizers while neglecting the regular addition of potassium fertilizers [37]. Apart from freely available potassium chemical fertilizer, potassium solubilizing bacteria release potassium from insoluble mineral sources [38]. We have identified seventy-seven percent (n = 40/52) isolated strains to solubilize potassium. Among thirteen selected strains, only one strain *A*. *kerstersii* (DZ 16) could not solubilize potassium.

**Fig 9:**
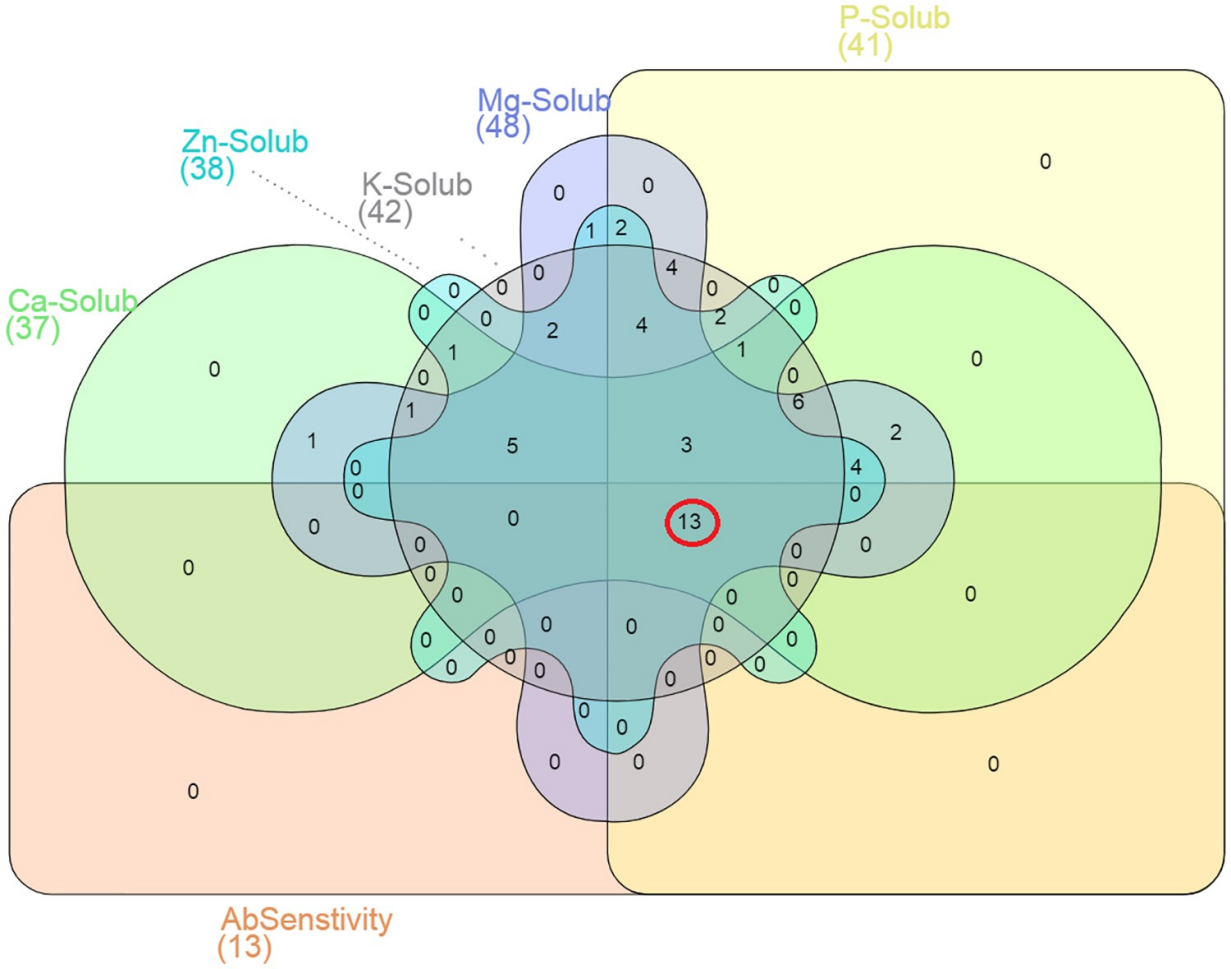
Venn diagram of antibiotic sensitive, phosphorous (P), potassium (K), calcium (Ca), magnesium (Mg) and zinc (Zn) solubilizers. Nutrient solubilization ability of identified bacterial strains (n = 52); P- solubilizers, K- solubilizers, Ca- solubilizers, Mg- solubilizers and Zn- solubilizers were sorted using Venn diagram. AbSensitivity (Antibiotic sensitivity), Ca-Solub (Calcium solubilization), Zn-Solub (Zinc solubilization), K-Solub (potassium solubilization), Mg-Solub (magnesium solubilization), P-Solub (phosphorus solubilization).

Comprehensive attempts were made for testing mineral solubilization (P-K-Ca-Mg-Zn) abilities of plant growth-promoting bacteria from bovine manure. We tested all isolates for their ability to solubilize calcium, magnesium, and zinc in-vitro and results reported according to halo zone formation. The Venn diagram sported nutrient solubilizing ability along with antibiotic susceptibility of isolates for six set shapes (Fig 9). Venn analysis showed that only 13 were positive for solubilization of all the aforementioned nutrients and sensitive to tested antibiotics in a plate assay. Among thirteen selected and sequenced strains to produce IAA and HCN, four strains belong to *Stenotrophomonas* genera, two strains to *Pseudomonas; s*ix belong to *Achromobacter* and only one to *Brevibacillus* genus. The sequence characterization using 16S rRNA analysis revealed these distinct genera previously not reported as plant growth-promoting bacteria from bovine dung samples.

**Fig 10:**
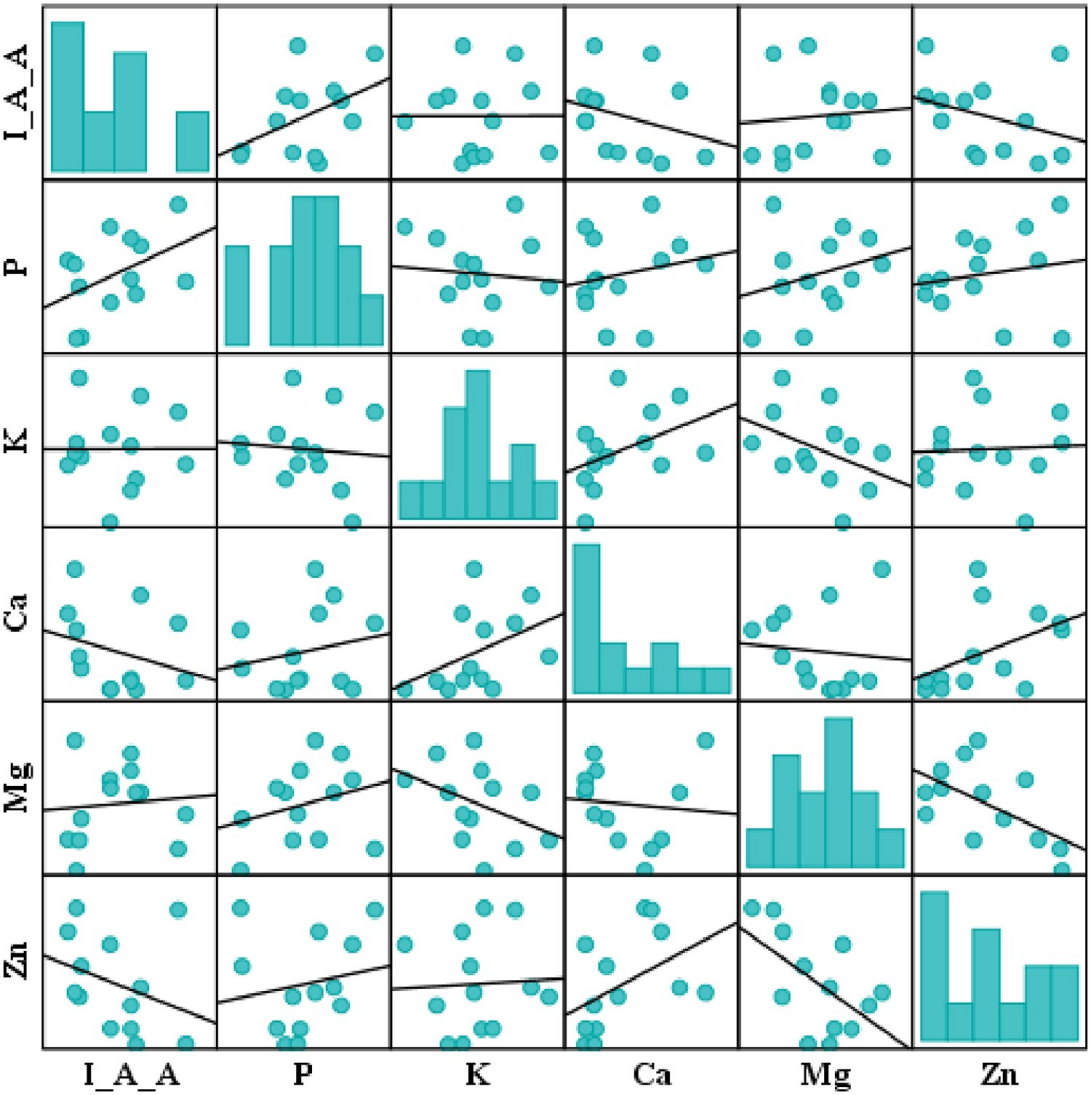
Pearson correlation analysis at significance value of p = 0.05. A diversified correlation was observed among quantified plant growth promotion tests i.e. IAA production and nutrient solubilization; phosphorous, potassium, calcium, magnesium and zinc.

Pearson correlation analysis showed a different relation between IAA, phosphorous, potassium, calcium, magnesium and zinc solubilization (Fig 10). IAA have a negative correlation with calcium (r = -0.28) and zinc (r = -0.32), a weak positive correlation with magnesium (r = 0.09), too weak positive correlation with potassium (r = 0.002). Phosphorous have a weak negative correlation with potassium (r = -0.09) and a positive correlation with calcium (r = 0.21), magnesium (r = 0.28) and zinc (r = 0.18). Potassium has a negative correlation with magnesium (r = -0.4) while a positive correlation with calcium (r = 0.42) and a weak positive correlation with zinc (r = 0.05). Calcium solubilization have a weak negative correlation with magnesium (r = 0.09) and positive with zinc (r = 0.45) while magnesium solubilization have a negative correlation with zinc solubilization (r = -0.58).

The thirteen strains used for radish germination in vitro and revealed that the germination rate was highest (46%) when seeds treated with DZ.15. Most of them are plant growth-promoting except DZ.2, as it has a deleterious affect i.e. 100% decrease for radish seed germination (Fig 7). The remaining 12 bacterial isolates showed promising results as plant growth-promoting bacteria for sustainable agriculture.

## Materials and methods

### 1. Sampling of bovine dung and isolation of bacterial strains

Healthy domestic bovine animals such as water buffalo, cow, ox and calf selected for sampling. Fresh dung samples from each animal dung pat, collected from different localities of Lahore and Khanqah Dogran, Punjab. A spatula full sample was taken from the moist interior of the dung pat in a sterile flask and delivered to the laboratory. One gram of the fresh fecal sample was taken and suspended in 10ml sterile distilled water (10%) by vortexing for 2 to 5 minutes. After mixing serial dilution technique [39] performed by taking 1ml from sample mix to 10^−1^ through 10^−6^ in sterile distilled water, for the isolation of culturable bacteria from each sample on nutrient agar. After the spreading, plates incubated at 37°C for 24 hours. After incubation, selected distinct colonies re-streaked on nutrient agar plates for purification. Microbial count recorded as CFU per gram of moist sample, well-distinguished colonies selected and stored as 30% glycerol stocks.

### 2. Metabolic profile of isolated bacterial strains

#### a. Nitrogen fixation

Isolated bacterial strains evaluated for their presumptive ability of nitrogen fixation using previously reported selective medium i.e. nitrogen-free mannitol agar [40]. Purified strains were inoculated on the NFM medium and incubated at 37°C for 2–3 days or up to seven days for visible growth of bacterial strains.

#### b. Auxin production

Isolated bacterial strains were tested for IAA production by colorimetric method using yeast extract mannitol medium [41] supplemented with L-tryptophan and results were evaluated using Salkowski’s reagent. Bacterial strains were first grown in yeast extract mannitol broth containing L-tryptophan for four days and cell-free supernatant was used for colorimetric detection of IAA by adding reagent at 535 nm. The amount of IAA produced by bacterial strains was estimated using a standard curve of IAA.

#### c. Hydrogen cyanide (HCN) production

Hydrogen cyanide (HCN) production by isolated strains was evaluated by Castric and Castric (1983) method [42]. Strains were swab inoculated on nutrient agar supplemented with 4.4g/L of glycine. A filter paper soaked in a solution of 2% NaCO_3_ and 0.5% picric acid was placed in the lid of the Petri plate. After incubation at 37°C for 4–7 days, filter paper was checked for change in color from yellow to orange brown due to HCN production.

#### d. Phosphorus and potassium solubilization

Solubilization of phosphorus and potassium by bacterial strains was evaluated using National Botanical Research Institute Phosphate (NBRIP) medium [43] and Aleksandrov medium [44], respectively. All bacterial strains were inoculated as spots on both media separately and incubated at 37°C for 5 to 7 days and examined for the formation of halo zones around inoculated culture. The formation of a clear zone indicates the solubilization of phosphorus and potassium by bacterial strains.

#### e. Calcium, magnesium and zinc solubilization

Calcium solubilization by isolated bacterial strains identified by using calcite [45] agar medium and magnesium solubilization examined using glucose agar plates supplemented with magnesium chloride. Bacterial strains spotted on respective plates and incubated at 37°C for 5 to 7 days. Calcium and magnesium solubilizing ability of isolated strains was examined by a clear zone formation around the bacterial growth. The solubilization of zinc evaluated by spotting on mineral salt agar supplemented with insoluble 0.1% ZnO and 1% glucose [46]. The plates were incubated for 7 to 10 days and observed for halo zones around bacterial growth.

### 3. Antibiogram test

Antibiogram test performed for five selected antibiotics (lowest disk concentrations). These antibiotic resistance breakpoints, as recommended by the CLSI for ampicillin (2 μg/ml), ceftriaxone (5 μg/ml), tetracycline (30 μg/ml), streptomycin (10 μg/ml) and erythromycin (5 μg/ml). Isolated bacterial strains tested for antibiotic susceptibility following Kirby-Bauer’s disc diffusion method [47].

### 4. Molecular characterization of selected bacterial strains

Thirteen strains selected based on the metabolic profile for molecular characterization by 16S rRNA sequencing. The DNA isolation was performed by using a Thermo Scientific Genejet genomic purification kit (catalog #K0721) followed by amplification using universal primers [48]. Amplified products sent to the Macrogen sequencing services of South Korea. The resulting sequences were identified by nucleotide BLAST from NCBI and deposited in GenBank for accession number. Phylogenetic analysis of sequenced strains for construction of phylogenetic tree was carried out using MEGA 7 software by maximum likelihood method of multiple sequence alignment tools at 100 Bootstrap values as several data sets.

### 5. Radish germination

A single colony of purified bacterial strain inoculated in nutrient broth overnight and after incubation 1ml of culture centrifuged and washed twice with sterile distilled water (SDW). After washing the bacterial pellet of each strain suspended in 600μL of sterile distilled water and used for seed treatment. Twenty radish seeds were surface sterilized using 5% NaOCl for one minute, washed three times with SDW and immersed in a bacterial suspension for 5 minutes with continuous shaking. For the control, seeds were incubated for 5 minutes in SDW accordingly. Bacterified seeds grown on sterilized filter paper-lined Petri dishes in wet conditions at room temperature. All Petri plates were incubated in the dark for germination up to 7 days and analyzed daily for the germination rate [49].

### 6. Statistical analysis

The results of all strains for auxin, HCN production, and putative nitrogen fixation analyzed by three-set Venn diagram for sorting in a common group having strains with these three PGP traits. Nutrient solubilization capabilities along with antibiotic susceptibility further analyzed by the Venn diagram of six sets for sorting in a common group with solubilization ability and antibiotic sensitive strains. Thirteen strains sorted by six sets Venn diagram used for radish germination. Statistical software SPSS used to analyze quantitative IAA production and nutrient solubilizing indexes by Pearson’s correlation of selected 13 strains, at significance value (α = 0.05). Correlation analysis applied to find a relationship among these hypothesized plant growth promotion parameters. Radish germination results analyzed using ANOVA and DMRT to compare the effect of bacterial strains on seed germination. All statistical experiments performed at a significant *P* value (α = 0.05).

## Conclusion

In this study, bacteria isolated from local breeds of different bovine animal have shown potential to be used as plant growth promoting bacteria. In-vitro tests used for plant growth promotion were production of IAA, HCN, presumptive nitrogen fixation, solubilization potential for phosphorous, potassium, calcium, magnesium and zinc. Sequence characterization using 16S rRNA analysis revealed three distinct genera previously not reported as plant growth promoting bacteria in particular. All sorted bacteria (n = 13) except DZ.2, have potential for plant experiments as biofertilizer. Further studies should evaluate their potentials for plant microbe interaction and suitable experiment host. Furthermore, our study highlights the disadvantage of unmonitored use of manure and its microbiota which may contain antibiotic resistant bacteria and potential unexplored plant pathogens. Therefore, we suggest that bovine manure flora should be explored for their potentials as biofertilizers.

## Abbreviations

Ca: Calcium
HCN: Hydrogen Cyanide
IAA: Indole Acetic Acid
K: Potassium
Mg: Magnesium
N: Nitrogen
NBRIP: National Botanical Research Institute Phosphate medium
NF: Nitrogen Fixation
NFM: Nitrogen Free Mannitol agar
P: Phosphorus
PGP: Plant Growth Promotion
PGPB: Plant Growth Promoting Bacteria
Zn: Zinc
